# The arterial–venous continuum: bridging atherosclerosis and chronic venous disease through endothelial dysfunction

**DOI:** 10.1007/s11739-026-04351-9

**Published:** 2026-04-09

**Authors:** Elena Campello, Andrea Boccatonda, Angela Napolitano, Chiara Simion, Paolo Simioni

**Affiliations:** 1 First Chiar of Internal Medicine and Thrombotic and Hemorrhagic Diseases Unit, Department of Medicine, University Hospital of Padova, 35128 Padua, Italy; 2 Diagnostic and Therapeutic Interventional Ultrasound Unit, IRCCS Azienda Ospedaliero-Universitaria di Bologna, Policlinico Sant’Orsola-Malpighi, Via Massarenti N 9, 40138 Bologna, Italy

**Keywords:** Atherosclerosis, Chronic venous disease, Chronic venous insufficiency, Endothelial dysfunction, Inflammation, Arterial stiffness, Cardiovascular risk, Peripheral artery disease

## Abstract

Atherosclerosis and chronic venous disease are among the most common vascular disorders worldwide and frequently coexist in the same individuals. Although traditionally regarded as distinct entities—one arterial and lipid-driven, and the other venous and pressure-related—emerging evidence suggests that they may share systemic determinants and overlapping biological pathways. This narrative review examines epidemiological and mechanistic evidence supporting potential links between atherosclerosis and chronic venous disease, and discusses implications for integrated vascular risk assessment. Observational studies report associations between the presence and clinical severity of chronic venous disease and a higher prevalence of atherosclerotic cardiovascular disease, peripheral artery disease, cerebrovascular disease, and increased all-cause mortality. Mechanistic research identifies converging processes—including systemic inflammation, oxidative stress, reduced nitric oxide bioavailability, extracellular matrix remodeling, and endothelial dysfunction—that affect both arterial and venous territories. Hemodynamic interactions between the arterial and venous systems may contribute to microvascular dysfunction and tissue hypoxia. Large-scale population analyses have also described associations between varicose veins and cognitive decline, including vascular dementia; however, these findings derive from observational data and do not establish causality. The concept of an arterial–venous continuum provides a biologically plausible and hypothesis-generating framework for understanding shared vascular vulnerability. Nevertheless, current evidence remains predominantly observational, and chronic venous disease should be regarded primarily as a potential marker of systemic endothelial dysfunction rather than a proven causal mediator of atherosclerotic progression. Further longitudinal and interventional studies are needed to clarify directionality and determine whether treatment of venous disease influences arterial outcomes.

## Introduction

Atherosclerosis is a chronic, lipid- and inflammation-driven disease of medium- and large-sized arteries, characterized by endothelial dysfunction, foam cell formation, fibrous cap development, and plaque complications, such as thrombosis and rupture [1, 2]. Chronic venous disease comprises a spectrum ranging from telangiectasias and varicose veins to chronic venous insufficiency (CVI) with edema, skin changes, and venous ulceration (CEAP C0–C6) [3].

Although these conditions affect different vascular territories and have traditionally been evaluated separately, patients with venous disease frequently exhibit clusters of traditional cardiovascular risk factors, low-grade systemic inflammation, and biomarkers of vascular dysfunction [4].

The emerging concept of a unified “vascular continuum” proposes that venous hemodynamic abnormalities and inflammation may trigger microvascular damage and endothelial activation that can promote arterial atherogenesis, and vice versa [2, 5]. Recent mechanistic studies highlight microvascular injury, oxidative stress, and endothelial activation as central processes linking these diseases [5, 6].

The clinical relevance of this overlap is substantial. If chronic venous disease also identifies individuals at higher risk for atherosclerotic events, systematic cardiovascular prevention strategies could be intensified in this population [7, 8]. Conversely, optimal control of atherosclerotic risk factors may reduce venous inflammation and edema, potentially alleviating symptoms of venous disease [2, 8].

In this review, we summarize epidemiologic evidence linking chronic venous disease and atherosclerotic outcomes, describe shared mechanisms—including inflammation, endothelial dysfunction, oxidative stress, arterial stiffness, and a prothrombotic milieu—and discuss implications for integrated vascular care.

## Epidemiologic evidence linking atherosclerosis and chronic venous disease

Historical cohort data first hinted at a potential arterial–venous link [4] (Table 1). In the Paris Prospective Study, middle-aged French men aged 42–53 years were followed for an average of 6.6 years [4]. Those with clinically evident varicose veins showed a higher incidence of intermittent claudication and “hard” coronary heart disease events, particularly among individuals in lower socioeconomic classes, whereas no association was seen with angina [4]. Subsequent population-based investigations provided quantitative support for these associations [9]. In a cross-sectional study of 902 women aged 45–54 years, symptoms consistent with chronic venous disease were present in 67.2% of participants, with 12.6% reporting severe symptoms [9]. The prevalence of abnormal ankle–brachial index (ABI < 0.9)—a marker of peripheral artery disease (PAD) and generalized atherosclerosis—was significantly higher in women with any venous symptoms (*p* = 0.005) and in those with severe symptoms (*p* = 0.029) [9]. Women with symptomatic chronic venous disease also exhibited a greater burden of cardiovascular risk factors, including higher BMI, waist circumference, triglycerides, C-reactive protein (CRP), and lower HDL cholesterol [9].

**Table 1:** Core shared mechanisms linking atherosclerosis and chronic venous disease

Core mechanism	Key pathophysiological features	Arterial manifestation	Venous manifestation	Clinical implications
Endothelial dysfunction	Reduced NO bioavailability; increased permeability; adhesion molecule expression; procoagulant shift	Plaque initiation progression impaired vasodilation	Venous hypertension valve dysfunction microvascular inflammation	Central integrative mechanism; potential target for global vascular risk assessment
Inflammation and oxidative stress	Cytokine activation (IL-6 TNF-α); ROS overproduction; NF-κB signaling; glycocalyx disruption	Atherogenesis plaque instability	Venous wall remodeling ulcer formation	Shared redox–inflammatory substrate across vascular beds
Extracellular matrix remodeling (MMPs)	MMP activation; collagen degradation; structural wall changes	Plaque destabilization arterial stiffness	Vein dilation valve incompetence post-thrombotic remodeling	Structural driver of vascular progression
Prothrombotic milieu	Tissue factor upregulation; platelet activation; impaired fibrinolysis (PAI-1 TAFI); endothelial–platelet crosstalk	Atherothrombosis (MI stroke)	VTE valve injury thrombus persistence	“Common soil” linking arterial and venous thrombotic events

The table summarizes the principal pathophysiological pillars underlying the proposed arterial–venous continuum.

These mechanisms are supported by converging epidemiological translational and experimental evidence and represent the central drivers of systemic vascular vulnerability

*IL* interleukin, *MI* myocardial infarction, *MMP* matrix metalloproteinase, *NF-κB* nuclear factor kappa B, *NO* nitric oxide, *PAI-1* plasminogen activator inhibitor-1, *ROS* reactive oxygen species, *TAFI* thrombin-activatable fibrinolysis inhibitor, *TNF-α* tumor necrosis factor alpha, *VTE* venous thromboembolism

Patients with advanced chronic venous disease exhibit higher pulse wave velocity (PWV) and indices of arterial stiffness compared with controls, independently of age and blood pressure [10]. Increased arterial stiffness augments pulsatile load and may exacerbate microvascular damage in dependent tissues, whereas chronic venous hypertension can, in turn, alter arterial inflow through changes in microvascular resistance [10].

Contemporary large-scale cohort analyses strengthened and quantified these observations. In the Gutenberg Health Study [11], approximately 15,000 adults aged 35–74 years were followed for a mean of 6.4 years. After adjustment for age, sex, and conventional cardiovascular risk factors, individuals with CVI had significantly higher morbidity and mortality: all-cause mortality hazard ratio (HR) was 1.52 (95% CI 1.24–1.86), and remaining robust after extensive adjustments (HR 1.46 [1.19–1.79]) [11]. Cardiovascular morbidity was also elevated (prevalence ratio (PR) 2.28 [1.75–2.97]) among those with symptomatic CVI compared with asymptomatic individuals [11]. The prevalence of PAD rose from 1.6% in CEAP C0–C1 to 9.9% in C4–C6 stages, and venous thromboembolism (VTE) from 2.6 to 11.0%, demonstrating a clear dose–response by clinical severity [11].

Beyond classical cardiovascular endpoints, the vascular overlap may extend to the brain. In a nationwide cohort study of 430,875 individuals from the Korean NHIS-HEALS database, of whom 5,096 (1.3%) had varicose veins, participants were followed for a median of 13.3 years [12]. The presence of varicose veins was associated with a 23% higher risk of all-cause dementia (HR 1.235 [1.147–1.329]) after propensity-score matching and multivariable adjustment [12]. Notably, individuals who underwent varicose vein treatment showed a lower risk of vascular dementia (HR 0.566 [0.382–0.841]), suggesting that effective management of venous disease may mitigate cerebrovascular microangiopathy, although this finding may reflect residual confounding or healthcare-related factors, and does not establish a causal protective effect of venous interventions [12]. Subgroup analyses revealed stronger associations in men, current smokers, and heavy drinkers [12].

Moreover, a recent umbrella review and meta-analysis synthesized 20 observational studies (≈ 393,875 participants, 55,356 cardiovascular events) and found that 17 of 20 investigations reported significant associations between chronic venous disease and at least one cardiovascular outcome (coronary artery disease, stroke, PAD, heart failure, or mortality) [8]. Pooled estimates indicated an overall effect size ranging from 1.3 to 3.8, strongest in CEAP C3–C6 stages, though heterogeneity in disease definitions remained high [8].

Furthermore, the coexistence of arterial and venous thrombosis is now increasingly recognized [13]. Clinical data indicate that up to 15–20% of patients with venous thromboembolism (VTE) experience concurrent or sequential arterial thromboembolic events—predominantly myocardial infarction or ischemic stroke [13]. Conversely, individuals with established atherosclerotic cardiovascular disease (ASCVD) exhibit an elevated risk of subsequent VTE. In a large prospective population analysis, the risk of arterial events after VTE was especially pronounced in younger individuals: HR 3.28 (1.69–6.35) in women and 2.06 (1.32–3.20) in men aged < 65 years, although the population-attributable fraction remained modest (≈ 0.9%) [14]. Following myocardial infarction, the risk of secondary VTE is transiently high—up to 23-fold within the first 30 days—and then declines over subsequent months, particularly affecting pulmonary embolism [14, 15].

## Shared pathophysiological mechanisms

**Fig. 1:**
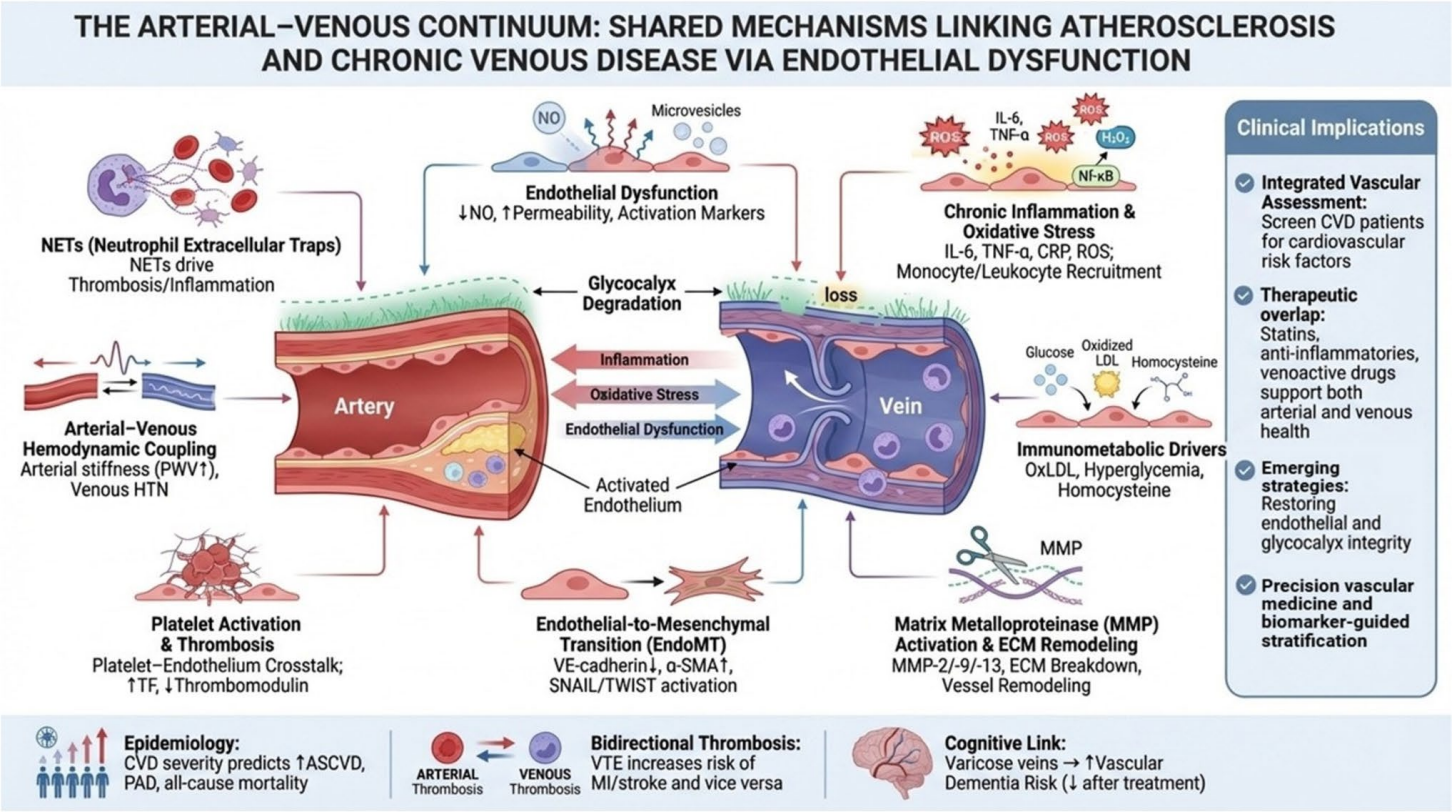
Conceptual overview of the shared mechanisms linking atherosclerosis and chronic venous disease along an arterial–venous continuum. Endothelial dysfunction represents the central integrative pathway, driven by inflammation, oxidative stress, and glycocalyx degradation, leading to leukocyte recruitment, platelet activation, and thromboinflammation in both arterial and venous beds. Extracellular matrix remodeling, endothelial-to-mesenchymal transition, immunometabolic alterations, and arterial–venous hemodynamic coupling contribute to vascular remodeling and bidirectional arterial and venous thrombosis, supporting a unified view of systemic vascular vulnerability with implications for integrated cardiovascular and venous risk assessment. *ASCVD* atherosclerotic cardiovascular disease, *CRP* C-reactive protein, *ECM* extracellular matrix, *ED* endothelial dysfunction, *IL* interleukin, *LDL* low-density lipoprotein, *NF-κB* nuclear factor-κB, *NO* nitric oxide, *OxLDL* oxidized low-density lipoprotein, *PAD* peripheral artery disease, *PWV* pulse wave velocity, *ROS* reactive oxygen species, *TF* tissue factor, *TNF-α* tumor necrosis factor-α, *VTE* venous thromboembolism

Several interconnected biological pathways plausibly link chronic venous disease with atherosclerosis [5, 8, 16] (Fig. 1). These bidirectional hemodynamic alterations support the concept of a functional coupling between arterial and venous systems, whereby venous pathology can influence arterial tone and vice versa. Molecular mechanisms have been organized into a structured hierarchy distinguishing primary integrative pathways (Table 1) from secondary supportive mechanisms (Table 2) and emerging experimental regulators (Table 3).

**Table 2:** Emerging redox- and thromboinflammatory mechanisms supporting the arterial–venous continuum

Mechanism	Experimental/clinical model	Key molecular pathway	Main findings	Quantitative/directional effect	Translational relevance
RBC-derived microvesicles	Human VTE cohorts; in vitro platelet assays	Phosphatidylserine exposure; TF carriage; NO scavenging	RBC-derived EVs enhance thrombin generation and platelet activation	Increased thrombin generation; association with thrombotic burden	Mechanistic; no therapeutic targeting yet
Iron–hepcidin axis	Human VTE patients; inflammatory models	IL-6 → JAK2/STAT3 → Hepcidin ↑; Fenton reaction	Iron overload amplifies ROS and endothelial injury	Higher hepcidin and lipid peroxidation markers in VTE	Potential biomarker; therapeutic modulation unproven
Ferroptosis (TFRC pathway)	Rat DVT model	TFRC–THBS1–CD47 axis; lipid peroxidation	TFRC knockdown reduces thrombus size and oxidative damage	Reduced thrombus burden in treated animals	Preclinical only
TXNIP–NLRP3 axis	Experimental DVT model	TXNIP inhibits thioredoxin → ROS ↑ → NLRP3 activation	TXNIP silencing decreases thrombus formation and IL-1β release	Reduced inflammatory infiltration and thrombus weight	Experimental; inflammasome inhibition under investigation
Platelet mitochondrial regulation (MTH1)	Murine thrombosis model	mtDNA oxidation control; ATP/calcium signaling	MTH1 deficiency impairs platelet activation	Reduced aggregation and thrombus formation	Mechanistic; not clinically targeted
Polyhedrocytes	Ex vivo clot contraction; MI and PE thrombi imaging	RBC compression during fibrin contraction	Polyhedral RBC packing reduces clot permeability	Increased clot resistance to fibrinolysis	Structural insight; not therapeutically targeted
Epigenetic modulation (SIRT1/SIRT3; FOXO3)	Coronary microcirculation models	Mitochondrial ROS regulation; eNOS coupling	Reduced sirtuin activity associated with endothelial dysfunction	Increased oxidative signaling	Exploratory; not clinically validated

The table summarizes selected experimental and clinical pathways that further modulate oxidative stress endothelial dysfunction and thrombogenesis across arterial and venous territories.

These mechanisms are primarily supported by preclinical or exploratory human data and should be interpreted as supportive rather than core drivers of the arterial–venous continuum

*AP-1* activator protein-1; *ATP* adenosine triphosphate; *CD47* cluster of differentiation 47; *DVT* deep vein thrombosis; *eNOS* endothelial nitric oxide synthase; *EVs* extracellular vesicles; *FOXO3* forkhead box O3; *HIF-1 *hypoxia-inducible factor-1; *IL* interleukin; *JAK2* Janus kinase 2; *MI *myocardial infarction; *MTH1* MutT homolog 1; *mtDNA* mitochondrial DNA; *NF-κB* nuclear factor kappa B; *NO* nitric oxide; *NOX* NADPH oxidase; *PE* pulmonary embolism; *ROS* reactive oxygen species; *SIRT1/3* sirtuin 1 and 3; *STAT3* signal transducer and activator of transcription 3; *TF* tissue factor; *TFRC* transferrin receptor 1; *THBS1* thrombospondin-1; *TXNIP* thioredoxin-interacting protein; *VTE* venous thromboembolism

**Table 3:** Emerging mechanosensitive and epigenetic regulators of endothelial plasticity within the arterial–venous continuum

Pathway	Experimental/clinical context	Core molecular axis	Main biological effect	Vascular setting	Level of evidence
Piezo1–KLF2	Varicose veins; oscillatory shear stress models	Mechanosensing → KLF2 signaling ↓	Promotes EndMT and inflammatory activation under disturbed flow	Venous and atherosclerosis-prone arterial regions	Translational + experimental
DLL4–NOTCH1	Atherosclerosis-prone regions (LOSS models)	NOTCH signaling activation	Partial EndMT and endothelial inflammation	Primarily arterial; hypothesized venous relevance	Experimental
BMP4–SMAD5	Disturbed venous shear stress; human varicose veins	BMP4-dependent mechanotransduction	Flow-induced EndMT and venous wall remodeling	Venous	Translational + experimental
H19/TET1–TGF-β axis	Hyperglycemia and inflammatory models	Epigenetic demethylation → TGF-β signaling ↑	Endothelial reprogramming and fibrosis	Arterial; potential venous relevance	Experimental
miR-214/c-Ski	Ang II-induced endothelial stress	miRNA-mediated suppression of c-Ski	Enhanced TGF-β–dependent EndMT	Arterial hypertensive models	Experimental
miR-296-5p/S100A4	Deep vein thrombosis models	miRNA downregulation → S100A4 ↑	Endothelial apoptosis and thrombus formation	Venous	Experimental

The table summarizes selected experimental pathways linking disturbed flow epigenetic modulation and endothelial-to-mesenchymal transition (EndMT) in arterial and venous disease

These mechanisms are supported primarily by preclinical or translational data and should be interpreted as emerging modulators rather than established drivers of systemic vascular remodeling

*Ang II* angiotensin II, *BMP4* bone morphogenetic protein 4, *DLL4* Delta-like ligand 4, *EndMT* endothelial-to-mesenchymal transition, *KLF2* Krüppel-like factor 2, *LOSS* low oscillatory shear stress, *miR* microRNA, *SMAD* mothers against decapentaplegic homolog, *TGF-β* transforming growth factor-beta

### Core mechanisms

#### Endothelial dysfunction

Endothelial dysfunction (ED) serves as a key integrator of vascular injury in both atherosclerosis and chronic venous disease [17–19], thus leading to platelet adhesion, leukocyte recruitment, and vascular smooth muscle proliferation with vascular remodeling, driving both arterial plaque formation and venous remodeling [5, 20, 21]. In venous hypertension, increased shear stress and hydrostatic pressure promote endothelial activation, reduced nitric oxide (NO) bioavailability, and increased vascular permeability [21]. Elevated asymmetric dimethylarginine (ADMA) and circulating endothelial microparticles (EMPs) reflect impaired NO signaling and ongoing endothelial injury. Biomarkers, such as endocan (ESM-1) and endoglin (CD105), may indicate systemic endothelial stress, although their clinical utility remains investigational [5, 22]. Chronic venous hypertension also induces tissue hypoxia and vascular endothelial growth factor (VEGF)-mediated remodeling, leading to intimal hyperplasia, smooth muscle migration, and extracellular matrix deposition—structural alterations that parallel early atherosclerotic changes [20].

Circulating cellular markers further support a systemic endothelial imbalance. Increased EMPs and circulating endothelial cells (CECs) indicate activation and prothrombotic transformation, whereas reduced endothelial progenitor cell (EPC) and endothelial colony-forming cell (ECFC) function reflects impaired vascular repair capacity [5, 23].

MicroRNAs (miRNAs) have emerged as key epigenetic regulators of vascular inflammation, oxidative stress, and endothelial integrity. miR-126, miR-155, and miR-195 have been shown to modulate endothelial function, while miR-223 and miR-96 can influence platelet reactivity [24, 25]. Additional miRNAs, including miR-34a, miR-200c, and miR-146a, can modulate oxidative and inflammatory signaling, thereby linking epigenetic control to redox imbalance in chronic venous disease [26].

The endothelial glycocalyx—a carbohydrate-rich layer that maintains vascular permeability and antithrombotic balance—is disrupted in both atherosclerosis and venous disease. Its degradation exposes adhesion molecules, such as vascular cell adhesion molecule-1 (VCAM-1) and intercellular adhesion molecule-1 (ICAM-1), promoting leukocyte and platelet adhesion. Oxidative and mechanical stress exacerbates glycocalyx loss in venous hypertension, further amplifying microvascular inflammation [27].

#### Inflammation and oxidative stress

Both atherosclerosis and chronic venous disease are characterized by persistent low-grade inflammation and oxidative stress, which drive ED and vascular remodeling [28, 29]. In both vascular beds, leukocyte activation and redox imbalance disrupt the endothelial glycocalyx, increase vascular permeability, and promote tissue injury [30]. Elevated circulating cytokines—including interleukin (IL)-6, tumor necrosis factor (TNF)-*α*, and IL-1*β*—as well as increased reactive oxygen species (ROS), reflect systemic inflammatory activation largely mediated by nicotinamide adenine dinucleotide phosphate (NADPH) oxidase pathways [31]. In venous hypertension, leukocyte trapping and hypoxia amplify NF-κB signaling, matrix metalloproteinase activation, and extracellular matrix degradation—processes that parallel early atherogenic remodeling [32].

Oxidative stress impairs endothelial nitric oxide synthase (eNOS) activity, reduces NO bioavailability, and shifts the endothelium toward a pro-inflammatory and prothrombotic phenotype [33–35]. The main enzymatic sources of ROS in varicose veins include NADPH oxidases (NOX1/NOX2), uncoupled nitric oxide synthases (iNOS/eNOS), and xanthine oxidase (XO), driving excessive superoxide and hydrogen peroxide generation [33]. Elevated markers of lipid and protein oxidation—malondialdehyde (MDA), thiobarbituric acid reactive substances (TBARS), and carbonylated proteins—confirm sustained redox imbalance in CD [33, 34].

Systemic antioxidant capacity is diminished, with reduced activity of superoxide dismutase (SOD), catalase (CAT), and glutathione peroxidase (GPx). Although local upregulation of these enzymes occurs in varicose tissue, it is insufficient to counteract ROS overproduction [21]. The reduction in total antioxidant capacity reinforces the contribution of impaired redox homeostasis to endothelial injury and inflammation [27, 33, 34]. Therefore, excessive ROS can damage the glycocalyx, increase permeability, and perpetuate cytokine release, thus establishing a self-sustaining inflammatory loop [21].

#### Extracellular matrix remodeling

Endothelial-to-mesenchymal transition (EndoMT) represents a maladaptive form of endothelial plasticity contributing to vascular remodeling in both arterial and venous disease [36]. Under chronic inflammatory and oxidative stress, endothelial cells lose junctional integrity and acquire mesenchymal features, promoting fibrosis, intimal thickening, and reduced vascular compliance [20]. Experimental and human studies indicate that persistent inflammatory signaling—particularly through NF-κB and TGF-*β* pathways—can drive endothelial phenotype switching in atherosclerotic plaques [37]. Similar hypoxia-driven and iron-associated inflammatory conditions have been described in advanced chronic venous disease, suggesting that EndoMT may represent a shared downstream response to sustained endothelial stress across vascular territories [37]. Rather than constituting an independent mechanism, EndoMT can be interpreted as a structural consequence of prolonged endothelial dysfunction within the arterial–venous continuum.

Matrix metalloproteinases (MMPs), particularly MMP-2 and MMP-9, are key mediators of extracellular matrix degradation and structural vascular remodeling in both chronic venous disease and atherosclerosis [38, 39]. In venous pathology, increased MMP activity contributes to wall dilation, valve dysfunction, and ulcer formation, whereas in arterial disease, it promotes plaque progression and instability. Beyond matrix turnover, MMPs participate in thromboinflammatory signaling across the arterial–venous continuum. Experimental data indicate that selected MMP isoforms (e.g., MMP-13) may enhance venous thrombogenesis through platelet–endothelial interactions, while MMP-9 has been implicated in early atherogenic remodeling [40, 41]. Genetic analyses support a causal link between MMPs and venous thrombosis. Using Mendelian randomization, Han et al. demonstrated that circulating MMP-12 levels are causally associated with increased VTE risk (OR 1.04, 95% CI 1.01–1.07; *p* = 0.001), reinforcing their role as a potential thrombotic effector and biomarker [42].

Clinically, elevated MMP levels correlate with vascular inflammation and adverse remodeling in both arterial and venous settings. Although pharmacologic modulation of MMP activity (e.g., with sulodexide or venoactive agents) may attenuate endothelial activation, robust evidence that targeting MMPs improves hard cardiovascular outcomes remains limited [43, 44]. Complementarily, Harbsmeier et al. performed the first systematic review of biomarkers associated with post-thrombotic syndrome (PTS) following deep vein thrombosis [45]. Among 46 biomarkers, only MMP-1, MMP-8, and tissue inhibitors of metalloproteinases (TIMP)-1 and TIMP-2 consistently correlated with PTS risk [45]. Collectively, MMPs represent dynamic mediators linking inflammation, structural remodeling, and thrombosis within the arterial–venous continuum.

#### Prothrombotic milieu

Inflammation-driven endothelial activation induces a prothrombotic milieu common to both arterial and venous diseases, involving platelet activation, tissue factor (TF) expression, and reduced fibrinolysis [46]. Disruption of the TF–thrombomodulin axis and upregulation of plasminogen activator inhibitor-1 (PAI-1) shift the endothelium toward a procoagulant phenotype, while excessive activation of thrombin-activatable fibrinolysis inhibitor (TAFI) further stabilizes thrombi in both VTE and atherothrombosis [27, 47].

Platelets act as central amplifiers of this process through thrombin signaling, endothelial crosstalk, and release of procoagulant microvesicles, thereby sustaining vascular inflammation and thrombus propagation [27, 48, 49].

Lipid-mediated mechanisms further modulate thromboinflammation; indeed, oxidized LDL enhances platelet activation and leukocyte recruitment, whereas HDL exerts counter-regulatory antithrombotic effects [50–52]. In low-shear, hypoxic venous environments, these pathways converge into an integrated immunothrombotic circuit linking endothelial dysfunction, metabolic dysregulation, and coagulation [53, 54]. Disturbed flow and local hypoxia activate endothelial NF-κB signaling, promoting monocyte TF expression, Weibel–Palade body release of von Willebrand factor (vWF) and P-selectin, and NET formation—all of which accelerate thrombin generation [55]. In this milieu, platelets undergo a glycolytic metabolic shift and shed procoagulant platelet microvesicles that may carry TF [56]; elevated circulating platelet-derived microvesicles have been associated with future VTE events [57]. Collectively, these processes define an immunothrombotic circuit in which inflammation, metabolism (glucose, lipids, and homocysteine), and coagulation are interdependent drivers of thrombus initiation and propagation [54, 56, 58].

Moreover, neutrophil extracellular traps (NETs) represent an important link between inflammation and thrombosis across arterial and venous territories [59]. NETs consist of extracellular chromatin fibers decorated with neutrophil-derived proteins that provide a scaffold for platelet adhesion, red blood cell entrapment, and activation of tissue factor and factor XII, thereby amplifying thrombin generation [60]. NET formation is driven by oxidative stress and inflammasome activation, reinforcing the previously described redox–inflammatory loop [61]. NET-rich thrombi have been identified in acute coronary syndromes, ischemic stroke, and venous thromboembolism, supporting their role as shared mediators of arterial and venous occlusion [61, 62]. Although experimental strategies targeting NET formation (e.g., peptidyl arginine deiminase-4 inhibition or DNase administration) have shown promise, their clinical applicability and safety remain to be established [59, 62].

### Secondary supportive mechanisms

#### Mitochondrial dysfunction and redox imbalance

Emerging transcriptomic and experimental data suggest that mitochondrial dysfunction and impaired redox regulation may further contribute to vascular injury across arterial and venous territories [64]. Mitochondrial integrity influences platelet activation and thrombus propagation. Experimental data indicate that oxidative nucleotide damage within platelets may modulate thrombotic responses, positioning mitochondrial redox balance as a potential regulatory mechanism [63]. These pathways reinforce the central role of oxidative stress but remain primarily mechanistic and exploratory [17, 64].

#### Erythrocyte contributions to thrombus propagation

Beyond oxygen transport, erythrocytes may amplify thromboinflammation under conditions of oxidative stress. RBC-derived microvesicles expose phosphatidylserine and tissue factor, providing procoagulant surfaces that enhance thrombin generation and interact with platelets and endothelial cells [65–67]. Hemolysis-related NO scavenging and increased blood viscosity in low-shear venous environments may further promote thrombus stability [20, 67].

#### Iron/hepcidin axis

Iron dysregulation has been linked to enhanced oxidative stress and thrombosis. Experimental models suggest that iron overload and ferroptosis-related pathways may promote endothelial injury and thrombus formation, while clinical studies associate altered hepcidin levels with venous thromboembolism risk [68, 69]. These findings support a potential redox–iron interface in vascular disease, although translational relevance remains under investigation.

##### TXNIP–NLRP3

The thioredoxin-interacting protein (TXNIP)-NOD-like receptor protein 3 (NLRP3) inflammasome axis represents another redox-sensitive pathway implicated in venous thrombosis. Experimental inhibition reduces thrombus burden and inflammatory activation, suggesting that inflammasome-mediated signaling may amplify oxidative vascular injury [70].

##### Polyhedrocytes

During clot contraction, erythrocytes adopt polyhedral shapes that reduce clot permeability and fibrinolysis susceptibility. This structural remodeling, observed in both arterial and venous thrombosis, may contribute to thrombus persistence [71, 72].

### Emerging experimental regulators

Disturbed flow represents a key upstream trigger of endothelial plasticity across arterial and venous territories (Table 3). Experimental and translational studies indicate that altered shear stress may impair mechanosensitive signaling pathways, including the Piezo1–KLF2 axis, thereby promoting EndMT and inflammatory activation in both varicose veins and atherosclerosis-prone arterial regions [73] [21]. Additional mechanotransductive pathways, such as delta-like ligand 4 (DLL4)–notch receptor 1 (NOTCH1) and bone morphogenetic protein 4 (BMP4)-dependent signaling, have been implicated in flow-induced endothelial reprogramming, supporting the concept that oscillatory or low-shear stress favors maladaptive endothelial remodeling [74, 75]. However, those data remain largely experimental. Epigenetic mechanisms further modulate endothelial phenotype switching. Long noncoding RNAs and microRNAs (e.g., H19 and miR-214) may influence transforming growth factor-beta (TGF-*β*)-dependent signaling and endothelial plasticity under inflammatory or hypertensive conditions [76–78]. Although mechanistically informative, these pathways currently lack clinical validation and should be interpreted as emerging regulators rather than established drivers of the arterial–venous continuum.

## Clinical implications for integrated vascular care

The link between venous and arterial diseases carries important clinical implications [8]. Patients with chronic venous disease, particularly those categorized as CEAP C3–C6, should undergo comprehensive cardiovascular assessment, including evaluation of lipid profile, glycemia, blood pressure, inflammatory markers, smoking status, physical activity, and body mass index [8, 35].

Endothelial biomarkers (e.g., endocan and ADMA) have been proposed for risk stratification, but their routine clinical use remains investigational due to limited validation and lack of outcome-driven evidence [5]. Functional vascular assessments (e.g., flow-mediated dilation, PWV, and microcirculatory measures) may provide mechanistic insight into systemic vascular dysfunction; however, their role in routine risk stratification of chronic venous disease patients is not yet standardized [20, 27].

Therapeutic management should follow an integrated vascular prevention strategy centered on lifestyle optimization and evidence-based cardiovascular risk reduction [79]. Lifestyle and dietary interventions may reduce systemic oxidative stress and support vascular health, although vascular outcome data specific to chronic venous disease populations remain limited. Nutritional interventions—including adherence to a Mediterranean diet—have been shown to reduce circulating NOX2-derived peptides and F₂-isoprostanes, thereby mitigating vascular oxidative stress. Future translational studies should define the optimal equilibrium between antioxidant protection and physiological redox signaling necessary to preserve erythrocyte–endothelial homeostasis [20, 27]. Statins remain foundational for lipid control and overall cardiovascular protection, with recognized beneficial effects on endothelial function according to current cardiovascular prevention guidelines [80]. Anti-inflammatory therapies are under investigation in selected high-risk populations; however, their specific role in modifying venous disease progression or combined arterial–venous risk remains to be established.

In the context of chronic venous disease, venoactive agents and glycocalyx-targeted therapies may improve local endothelial function and venous symptoms [21]. Compounds, such as micronized purified flavonoid fraction (MPFF), diosmin, and sulodexide, exert anti-inflammatory and venotonic effects, and are effective for symptom control and ulcer management [20]. Therapeutic modulation of MMP activity could also be clinically feasible. Patients with symptomatic chronic venous disease exhibited higher MMP-9 (+ 17%) and VCAM-1 (+ 29%) levels in venous samples, inducing endothelial senescence and inflammation in vitro [44]. Sulodexide treatment for 2 months reduced MMP-9, IL-6, VCAM-1, and vWF, restoring endothelial homeostasis through glycocalyx repair and suppression of MMP activation [44]. Nevertheless, evidence that these interventions influence systemic atherosclerotic outcomes or long-term arterial risk is currently lacking [21].

Compression therapy remains the cornerstone for managing edema and preventing ulceration, while endovenous ablation, sclerotherapy, or surgical interventions relieve symptoms and enhance quality of life [79]. Although conclusive evidence that treating chronic venous disease lowers atherosclerotic event rates is lacking, improving venous hemodynamics and mobility may indirectly promote cardiovascular health and reduce systemic inflammation [6, 8].

Managing patients who present with both arterial and venous thrombosis remains one of the most complex and delicate tasks in vascular medicine [79]. The therapeutic dilemma lies in balancing the prevention of recurrent thrombosis against the heightened risk of bleeding that inevitably accompanies intensified antithrombotic therapy. Treatment should follow a patient-centered and individualized approach, shaped by the vascular territory involved, the temporal relationship between events, and each patient’s thrombotic and hemorrhagic risk profile. Dual pathway inhibition or DOAC-based regimens may benefit selected patients, while triple therapy should be limited to short periods after coronary interventions in accordance with the current ESC and international antithrombotic guidelines [13].

## Limitations

Despite growing interest in the arterial–venous continuum, several important limitations must be acknowledged. First, most of the available evidence derives from observational cohort studies, which preclude causal inference and remain vulnerable to residual confounding. Patients with chronic venous disease frequently share traditional cardiometabolic risk factors with atherosclerosis, making it difficult to disentangle shared risk profiles from true pathophysiological interdependence. Second, heterogeneity in chronic venous disease classification—particularly across CEAP stages—limits comparability among studies and may obscure stage-specific associations with cardiovascular outcomes. Differences in diagnostic criteria, imaging modalities, and outcome definitions further contribute to variability across cohorts. Third, the absence of adequately powered interventional trials prevents definitive conclusions regarding whether treatment of venous disease modifies arterial or cognitive outcomes. Observed associations between venous interventions and reduced vascular events may reflect confounding by healthcare access, disease severity, or unmeasured lifestyle factors. Finally, reverse causation cannot be excluded, as systemic vascular dysfunction may predispose to both arterial and venous pathology rather than one condition directly promoting the other. Prospective longitudinal studies with standardized phenotyping and mechanistic endpoints are required to clarify directionality and causal relationships.

## Conclusions and future directions

Future research should aim to integrate endothelial biomarkers, functional vascular imaging, and multi-omics approaches to better characterize systemic vascular vulnerability. Whether such strategies will meaningfully refine risk prediction or guide targeted interventions remains to be determined through prospective validation studies. Standardized endothelial phenotyping may help improve comparability across studies and clarify the clinical relevance of endothelial dysfunction within the arterial–venous continuum [5, 31]. However, biomarker-guided management cannot yet be recommended in routine practice due to the absence of outcome-driven evidence. Pharmacologic strategies targeting inflammation, coagulation, and endothelial stability represent promising research avenues, but their impact across both arterial and venous disease requires confirmation in adequately powered clinical trials [20, 27]. Regenerative approaches, including endothelial progenitor cell-based strategies, are conceptually appealing but remain experimental [5]. Further research is needed to establish safety, feasibility, and long-term efficacy before clinical translation can be considered.

## Data Availability

All data generated or analysed during this study are included in this published article.
